# Effects of *L*-arginine on gut microbiota and muscle metabolism in fattening pigs based on omics analysis

**DOI:** 10.3389/fmicb.2024.1490064

**Published:** 2024-11-11

**Authors:** Chengming Liu, Yiting Yang, Meng Wang, Wenyu Jiang, Yong Du, Ziling Hao, Lei Chen, Kangping Zhu, Bin Liu, Lili Niu, Ye Zhao, Yan Wang, Mailin Gan, Linyuan Shen, Li Zhu

**Affiliations:** ^1^State Key Laboratory of Swine and Poultry Breeding Industry, College of Animal Science and Technology, Sichuan Agricultural University, Chengdu, China; ^2^Key Laboratory of Livestock and Poultry Multi-omics, Ministry of Agriculture and Rural Affairs, College of Animal Science and Technology, Sichuan Agricultural University, Chengdu, China; ^3^Farm Animal Genetic Resources Exploration and Innovation Key Laboratory of Sichuan Province, Sichuan Agricultural University, Chengdu, China; ^4^Sichuan Dekon Livestock Foodstuff Group, Chengdu, China

**Keywords:** *L*-arginine, meat quality, physiological metabolism, 16S rRNA, metabolomics

## Abstract

**Introduction:**

*L*-arginine is an α-amino acid and a semi-essential nutrient of significant biological interest. It plays a role in influencing various aspects of animal meat traits, gut microbiota composition, and physiological metabolism.

**Methods:**

This study aimed to investigate the combined effects of *L*-arginine supplementation on gut microbiota composition and the metabolism of the longissimus dorsi muscle in fattening pigs. Eighteen Yorkshire commercial pigs were divided into two groups: a control group that received no supplements and a treatment group that was given 1% *L*-arginine for 52  days. The diversity and composition of microorganisms in the feces of the control (NC) and L-arginine (Arg) groups were analyzed by sequencing the 16S rRNA V3 -V4 region of the bacterial genome.

**Results:**

The findings indicated that *L*-arginine supplementation increased both the abundance and diversity of gut microbiota, particularly affecting the Firmicutes and Bacteroidetes phyla. KEGG enrichment analysis revealed significant changes in several metabolism-related pathways, including amino acid, carbohydrate, and lipid metabolism. Metabolomic analysis identified 85 differential metabolites between the arginine and control groups, with phospholipids ranking among the top 20. Additionally, functional predictions indicated an increased abundance in the glycerophospholipid metabolism pathway. Correlation analysis linked changes in gut microbiota to phospholipid levels, which subsequently influenced post-slaughter meat color and drip loss.

**Discussion:**

These results suggest that *L*-arginine supplementation positively impacts gut microbiota composition and the metabolic profile of the longissimus dorsi muscle in fattening pigs, with potential implications for meat quality.

## 1 Introduction

Arginine, with the chemical formula C₆H₁₄N₄O₂, typically appears as white rhombic or monoclinic lamellar crystals. It is classified into two types based on the position of the amino group in its structure: synthetic *D*-arginine and biologically occurring *L*-arginine. The latter, which is naturally synthesized in organisms, is the predominant form that exerts physiological effects. Arginine is not only a crucial raw material for protein synthesis but also serves as a precursor for the production of several important substances, including creatine, polyamines, and nitric oxide (NO). Research has demonstrated that *L*-arginine plays significant roles in various physiological processes, metabolism, and antioxidant defense mechanisms (Wu et al., 2009; Fathi et al., 2011; Dian-chao, 2012; Castro et al., 2019). For example, arginine is a key component of the urea cycle, where it undergoes a series of reactions to produce urea, which is then excreted in the urine. This process helps remove excess amino groups from the body, thereby maintaining amino acid balance and stabilizing nitrogen metabolism (Xiao-jun, 2007). Additionally, arginine is a crucial precursor for the synthesis of nitric oxide (NO), ornithine, citrulline, creatine, and guanidine-butylamine. It is the sole precursor for NO production in the body. Catalyzed by nitric oxide synthase, arginine is converted into nitric oxide and various intermediates. The nitric oxide produced plays a vital role in regulating cell physiological functions and metabolic processes, including blood pressure regulation, immune response, and apoptosis, by modulating cell signaling pathways (Kokai et al., 2022; Zhang L. et al., 2023; Sakai et al., 2024). In conclusion, arginine is crucial for regulating physiological metabolism in organisms, influencing multiple metabolic pathways and physiological processes (Clancy et al., 2017; Wang et al., 2018).

Meat quality is a complex economic trait primarily influenced by genetic and nutritional factors, while the effects of feeding management and environmental factors are comparatively minimal. Arginine, a semi-essential amino acid, impacts meat quality through signaling pathways such as Akt and mTOR, which play crucial roles in muscle development and overall meat composition (Ma et al., 2020; Dou et al., 2023). Studies have confirmed that arginine can influence meat quality traits by affecting muscle growth, altering muscle fiber structure, reducing fat deposition, and regulating nitrogen balance (Lc et al., 1984; Otto et al., 2003; Watford and Wu, 2018). Supplementing swine diets with 1% arginine has been shown to significantly enhance tenderness, thereby increasing the acceptability of male pork (Madeira et al., 2014). In studies on frozen pig longissimus dorsi muscles, *L*-arginine was found to increase the a-values of the muscle. It is hypothesized that this effect may be related to the antioxidant properties of arginine (Bao et al., 2021). Studies on lamb muscle have shown that basal dietary supplementation with 1% *L*-arginine significantly reduced shear force in the longissimus dorsi muscle and increased a-values in the biceps femoris muscle. These changes were associated with alterations in muscle fiber type transformation (Ma et al., 2015; Wang et al., 2022). The study results indicated that dietary arginine significantly increased the mRNA levels of genes associated with muscle fat synthesis. This modulation of lipid metabolism *in vivo* ultimately led to an improvement in the marbling score of pork (Hu et al., 2017).

The intestinal tract is the primary site for food digestion and nutrient absorption, where food undergoes both mechanical and chemical breakdown into small molecules like glucose, amino acids, and fatty acids. Additionally, the health of the intestinal tract is vital for the host’s physiological metabolism and immune regulation (Zhao et al., 2023). Recent studies have demonstrated that arginine can enhance animal growth, metabolism, antioxidant capacity, and immunity by modifying the intestinal microbial community. Dietary arginine supplementation has been shown to alter the intestinal microbial composition in broilers, fostering the growth of beneficial bacteria and accelerating their growth rate (Zhang B. et al., 2020). Dietary supplementation with arginine can also regulate the composition of the intestinal microbial community, boost the antioxidant capacity of the intestinal tract, and enhance the immunity of yellow-feathered broilers (Ruan et al., 2020). These findings suggest that arginine can regulate the structure and function of the intestinal microbiome, maintaining its stability and balance, which benefits the host’s intestinal health, immune function, and metabolic status. Therefore, a thorough investigation into the mechanisms by which arginine affects the intestinal microbiome could lead to the development of new nutritional strategies, enhancing intestinal health and improving disease prevention and treatment.

This study examined the effects of dietary arginine supplementation on the gut microbiota structure and metabolome in fattening pigs using 16S rRNA sequencing, untargeted metabolomics, and phenotypic traits measured at slaughter. The research aimed to understand how arginine supplementation influences gut microbiota composition and metabolome, and to explore the correlations between changes in the gut microbiota, metabolome, and phenotypic traits.

## 2 Materials and methods

### 2.1 Ethics

All experimental procedures detailed below were approved by the Animal Ethical and Welfare Committee of Sichuan Agricultural University, Chengdu, China (No. 2020202051).

### 2.2 Animals and sample collection

The animals used in this study were provided by a registered breeding company in Sichuan Province, China. All animals had free access to food and water and were housed under similar environmental conditions, with ambient temperatures ranging from 25 to 35°C. The diets met or exceeded the National Research Council (NRC, 2012) recommendations for crude protein, micronutrients, vitamins, and energy at different stages of production. A total of 18 Yorkshire commercial pigs (Dan line) with an average age of (175.26 ± 5.84) days were selected and randomly assigned to two groups (9 pigs per group): a control group without *L*-arginine (NC) and a group with *L*-arginine added to the diet (Arg). The pigs were fed a basal diet supplemented with 1% *L*-arginine (10 g *L*-arginine per kg of liquid feed). After 52 days of feeding, rectal feces were collected from all pigs within 10 min of slaughter, and the longest muscle from the back was collected within 20 min of slaughter. All samples were immediately stored in liquid nitrogen tanks and later frozen at −80°C in a refrigerator upon return to the laboratory for subsequent microbiome and metabolomics analysis.

### 2.3 Phenotypic trait data collection

In terms of growth performance, initial body weight (IBW) and final body weight (FBW) were recorded at the beginning and end of the experiment, respectively, while average daily gain (ADG) and net gain (NG) were calculated subsequently. Post-slaughter, carcass weight was recorded for determining the dressing percentage. Additionally, carcass length was documented. Backfat thickness (in mm) was calculated by averaging measurements taken at three regions on the right side of the carcass: the first rib, the last rib, and the last lumbar vertebra. The eye muscle area was measured at the last rib using vernier calipers. Measurements for pH at 45 min, meat color, drip loss, purge loss, and cooking loss were conducted using the longissimus dorsi (LD) muscle obtained from the left side of each carcass.

The pH and meat color parameters (L* lightness, a* redness, and b* yellowness) were assessed 45 min post-slaughter using a pH meter (pH-STAR, MATTHAUS, Germany) and a portable chromameter (CR-400, KONICA MINOLTA, Japan) respectively. Three measurements were taken at different areas of each chop, and the average value was calculated. Drip loss was assessed by weighing approximately 25 g of muscle after a 45-min postmortem period (W1). The muscle was then placed in a storage bag attached to a fishhook and kept at 4°C for 24 h. Afterward, the muscle was removed from the fishhook, carefully dried, and reweighed (W2). The drip loss value was calculated using the following formula: drip loss (%) = (W1 − W2)/W1 * 100. To determine the cooking loss, approximately 100 g of muscle was weighed (W3) and cooked in a steamer for 30 min. Following the cooking process, the muscle sample was promptly removed from the steamer, allowed to cool for 20 min at room temperature, and reweighed (W4). The cooking loss was calculated using the formula: cooking loss (%) = (W3 − W4)/W3 * 100.

### 2.4 16S rRNA sequencing and data analysis

Total genomic DNA was extracted using the CTAB/SDS method. DNA concentration and purity were verified by gel electrophoresis in 1% agarose gel. An aliquot of the sample was placed into a centrifuge tube and diluted to 1 ng/μL with sterile water. PCR amplifications were performed using the diluted genomic DNA as a template. The hypervariable V3–V4 region of the 16S rRNA gene was amplified using the primer pair 515F-806R (Caporaso et al., 2011). PCR amplification was performed using Phusion^®^ High-Fidelity PCR Master Mix (New England Biolabs, Ipswich, MA, United States) following the manufacturer’s instructions. PCR products were purified by agarose gel electrophoresis on 2% agarose gels. PCR products of between 400 and 450 bp were selected for PCR product purification using GeneJET (Thermo Scientific, Waltham, MA, United States) following the manufacturer’s instructions.

The 16S rRNA data were analyzed using QIIME2 (v2022.8.3) software and its integrated plugins. Initially, the QIIME tools import plugin was used to import single-end 16S rRNA sequencing data. Subsequently, the QIIME dada2 denoise-paired plugin and QIIME feature-table filter-features plugin were employed to denoise and filter the raw data. The filtered data were then used to construct a phylogenetic tree using the QIIME phylogeny align-to-tree-mafft-fasttree plugin. For *α*-diversity and *β*-diversity analysis, the QIIME diversity core-metrics plugin was utilized. Species annotation was performed using the QIIME feature-classifier plugin. Finally, functional prediction of OTUs abundance tables was carried out using PICRUSt2 (v2.5.2).

### 2.5 LC–MS/MS untargeted metabolomics sequencing

#### 2.5.1 Metabolite extraction

Weigh 20 mg of each sample, add 200 μL of pre-cooled 80% methanol water with 3 steel beads, homogenize and crush in a tissue crusher, then add 800 μL of pre-cooled 80% methanol water, vortex mixing, sonicate for 2 h in an ice bath, and then let stand overnight at −20°C. Centrifuge at 16,000 *g* at 4°C for 20 min, take the supernatant and add equal amounts of the internal standards Cholic acid-d4 and Cholic acid-d4 and ryptophan-d5 were added to the supernatant, and the supernatant was evaporated in a high-speed vacuum concentration centrifuge. The sample was redissolved in 50 μL of 80% methanol solution and centrifuged at 20,000 *g* for 15 min at 4°C, and the supernatant was prepared for mass spectrometry analysis.

#### 2.5.2 Mass spectrometry acquisition

Each sample was detected by electrospray ionization (ESI) in positive (+) and negative (−) modes, respectively. The samples were separated by UPLC and analyzed by mass spectrometry on a QTRAP 5500 mass spectrometer using a HESI source for ionization. The parameters of the QTRAP 5500 ESI source were as follows: Positive Ion Mode: Source Temperature 550°C, IonSource Gas1 (GAS1): 40, Ion Source Gas2 (GAS2): 50, Curtain Gas (CUR): 35, Ion Spray Voltage Floating (ISVF) 5,500 V. Negative Ion Mode: Source Temperature 550°C, Ion Source Gas1 (GAS1): 40, Ion Source Gas2 (GAS2): 50, Curtain Gas (CUR): 35, Ion Spray Voltage Floating (ISVF) −4500 V. MRM mode is used to detect the ion pairs to be measured.

#### 2.5.3 Data analysis

Metabolomic data preprocessing was initially conducted to generate a data matrix, which included retention time (RT), mass-to-charge ratio (m/z) values, and peak intensity. After annotating these metabolites, pathway analysis of the differential metabolites was performed using the LIPIDMaps^1^, HMDB^2^, and KEGG^3^ databases. Principal component analysis (PCA) and partial least squares discriminant analysis (PLS-DA) were carried out using metaX. Statistical significance (*p-*value) was computed through univariate analysis (*t*-test). Unless otherwise specified, the criteria for screening differential metabolites were VIP score > 1, *p-*value <0.05.

### 2.6 Statistical analysis

After organizing the raw experimental data into a matrix using Excel 2016, statistical analysis was performed using SPSS 26.0 (International Business Machines Corporation) and GraphPad Prism (v8.0) software. The data analysis involved one-way ANOVA and univariate statistical analysis. Results are presented as “mean ± standard deviation,” with significance determined by *p-*value <0.05 indicating statistical significance and *p-*value <0.01 indicating high statistical significance.

## 3 Results

### 3.1 Effect of *L*-arginine on growth performance, carcass traits and meat quality of fattening pigs

The basic daily feed nutritional composition used in the experiment is shown in Table 1. After 52 days of feeding, all pigs were slaughtered for data collection.

**Table 1:** The composition of basic diet and nutritional level.

Composition	Nutrients	Content (%)
Corn	Rough protein	≥14.0
Flour	Crude fiber	≤8.0
Wheat	Crude ash	≤8.0
Soybean meal	Calcium	0.40–1.20
Stone powder	Total phosphorus	≥0.40
Monocalcium phosphate	Sodium chloride	0.30–0.80
Sodium chloride	Lysine	≥0.65
Vitamins and vitamin-like substances	Water	≤13.0

The results indicate that arginine supplementation has a significant impact on the redness value (a) and drip loss of the Longissimus dorsi after slaughter. Specifically, the redness value (a) of the Longissimus dorsi in the arginine group is significantly higher than that in the control group, while the drip loss is significantly lower. Additionally, measurements of pressurized water loss, cooking loss, tenderness, and toughness reveal that arginine supplementation improves muscle tenderness, toughness, and hydraulic force of the Longissimus dorsi. However, apart from a significant reduction in carcass length, arginine supplementation does not notably affect other carcass traits or growth performance indicators (Table 2).

**Table 2:** Influence of arginine on growth, carcass, and meat quality traits of fattening pigs.

Group	Traits	Arg treatment	NC treatment	*P*-value
Growth Traits	IBW (kg)	89.32 ± 5.36	90.44 ± 5.16	0.654
	FBW (kg)	143.27 ± 9.37	150.63 ± 14.11	0.189
	NG (kg)	53.96 ± 11.09	60.19 ± 10.72	0.237
	ADG (g/d)	805.29 ± 165.51	898.32 ± 160.05	0.237
Carcass traits	Carcass weight (kg)	107.50 ± 7.39	113.63 ± 10.86	0.160
	Backfat thickness at three points (cm)	1.82 ± 0.62	2.10 ± 0.30	0.257
	Longissimus dorsi muscle area (cm^2^)	48.75 ± 3.36	51.95 ± 6.09	0.199
	Carcass length (cm)	105.32 ± 3.96	109.13 ± 2.52	0.029
	Carcass slant length (cm)	89.55 ± 3.01	91.88 ± 2.03	0.076
Meat quality traits	pH	6.06 ± 0.16	6.22 ± 0.24	0.099
	L*	44.05 ± 2.13	44.40 ± 1.44	0.687
	a*	5.04 ± 0.59	3.98 ± 0.66	0.002**
	b*	3.12 ± 0.89	3.42 ± 0.46	0.400
	Electric conductivity	2.88 ± 1.06	3.04 ± 1.45	0.775
	Pressurized water loss (%)	28.56 ± 2.63	30.52 ± 2.70	0.131
	Drip loss (%)	2.56 ± 1.45	4.56 ± 1.66	0.013*
	Cooking loss (%)	35.30 ± 2.60	37.71 ± 5.42	0.213
	Tenderness value (kg)	8.67 ± 3.10	8.27 ± 2.50	0.769
	Toughness value (kg.sec)	36.03 ± 15.79	32.92 ± 12.09	0.648

IBW, initial body weight; FBW, final body weight; NG, net gain; ADG, average daily gain. *P < 0.05, **p < 0.01, ***p < 0.001.

### 3.2 Effect of feeding arginine on the intestinal microbial community of fattening pigs

#### 3.2.1 Feeding *L*-arginine improves intestinal diversity and abundance in fattening pigs

**Figure 1:**
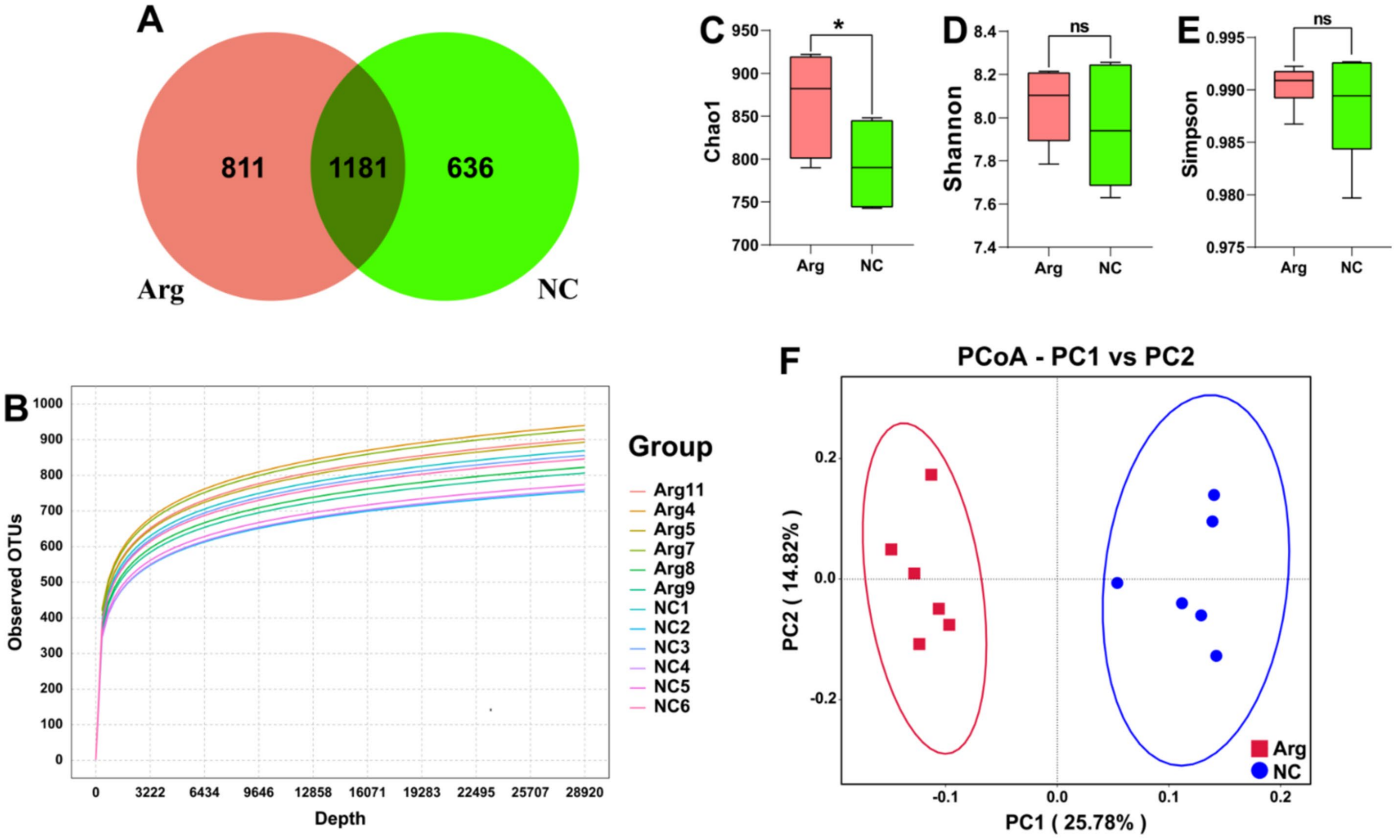
Quality and diversity analysis results of 16S rRNA sequencing data. **(A)** Venn diagram of OTU quantity distribution between *L*-arginine group and control group. **(B)** *α*-diversity rarefaction curve based on OTUs. **(C–E)** Chao1, Shannon, and Simpson indices results for *L*-arginine group and control group. **(F)** *β*-diversity PCoA plot based on UniFrac distances.

After quality control and clustering, a total of 2,682 OTUs are obtained. Among these, the arginine-treated group has 811 unique OTUs (30.23%), while the control group has 636 unique OTUs (23.71%). There are 1,181 OTUs shared between the two groups, accounting for 44.03% of the total OTUs (Figure 1A). The rarefaction curves based on OTUs show that the curves flatten out at the end, indicating sufficient sampling depth for individual samples (Figure 1B). Additionally, when plotted by groups, the rarefaction curves for the arginine-treated group lie above those of the control group, suggesting higher species diversity in the arginine-treated group (Supplementary Figure S1).

In the subsequent analysis of species diversity, *α*-diversity metrics indicate that the Chao1 index, reflecting species richness, is significantly higher in the arginine-treated group compared to the control group. However, no significant differences are observed in the Simpson index, which assesses species evenness, and the Shannon index, which provides a comprehensive measure of *α*-diversity (Figures 1C–E). In the *β*-diversity analysis, PCoA using unifrac distances reveals significant differences in species composition between the arginine-treated group and the control group (Figure 1F).

In summary, arginine feeding increases species richness within the gut microbiota of Yorkshire pigs and alters its structural composition. However, it has no impact on the evenness of the gut microbiota distribution in Yorkshire pigs.

#### 3.2.2 Feeding *L*-arginine alters the composition of intestinal flora in fattening pigs

**Figure 2:**
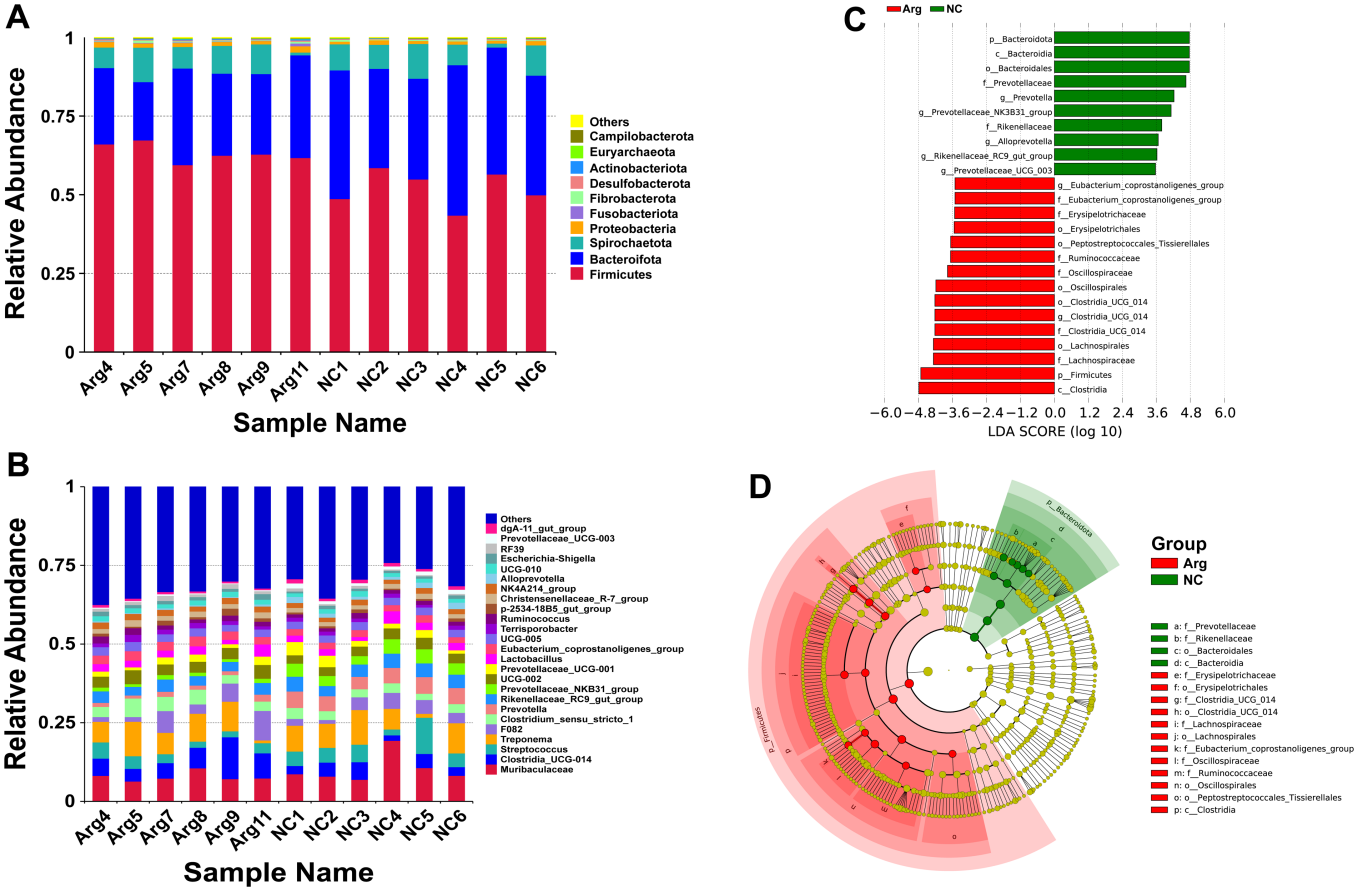
Species annotation and differential analysis of gut microbiota between *L*-arginine group and control group. **(A,B)** Stacked bar chart of species distribution at phylum and genus levels. **(C,D)** LEfSe differential analysis: LDA score distribution bar chart and phylogenetic tree.

Following arginine supplementation in the diet, the phylum-level species annotation results for the Yorkshire pig gut microbiome reveal that Firmicutes, Bacteroidetes, and Spirochaetes comprise over 95% of the microbiota. Among these, the abundance of Firmicutes significantly increases (63.32% vs. 52.00%), while the abundance of Bacteroidetes decreases (26.31% vs. 38.44%). The abundance of other phyla, including Spirochaetes, remains relatively unchanged (Figure 2A). At the genus level, arginine supplementation leads to a notable increase in the abundances of Clostridia_UCG-014 (7.05% vs. 3.61%) and Clostridium_sensu_stricto_1 (4.02% vs. 2.79%), while the abundances of Streptococcus (3.17% vs. 5.43%) and Prevotella (1.42% vs. 4.86%) significantly reduce (Figure 2B). LEfSe analysis indicates that the main differences in gut microbiota between the arginine-supplemented group and the control group concentrate within Bacteroidetes and Firmicutes. Within Bacteroidetes, genera such as Alloprevotella, Prevotella, Prevotellaceae_NK3B31_group, and Prevotellaceae_UCG_003 from the Prevotellaceae family, as well as Rikenellaceae_RC9_gut_group from the Rikenellaceae family, predominantly enrich in the control group. Conversely, within Firmicutes, genera such as Erysipelotrichaceae, Lachnospiraceae, Oscillospiraceae, Ruminococcaceae, and Clostridia_UCG-014 are more abundant in the arginine-supplemented group (Figures 2C,D).

#### 3.2.3 Feeding *L*-arginine affects the function of intestinal flora in fattening pigs

**Figure 3:**
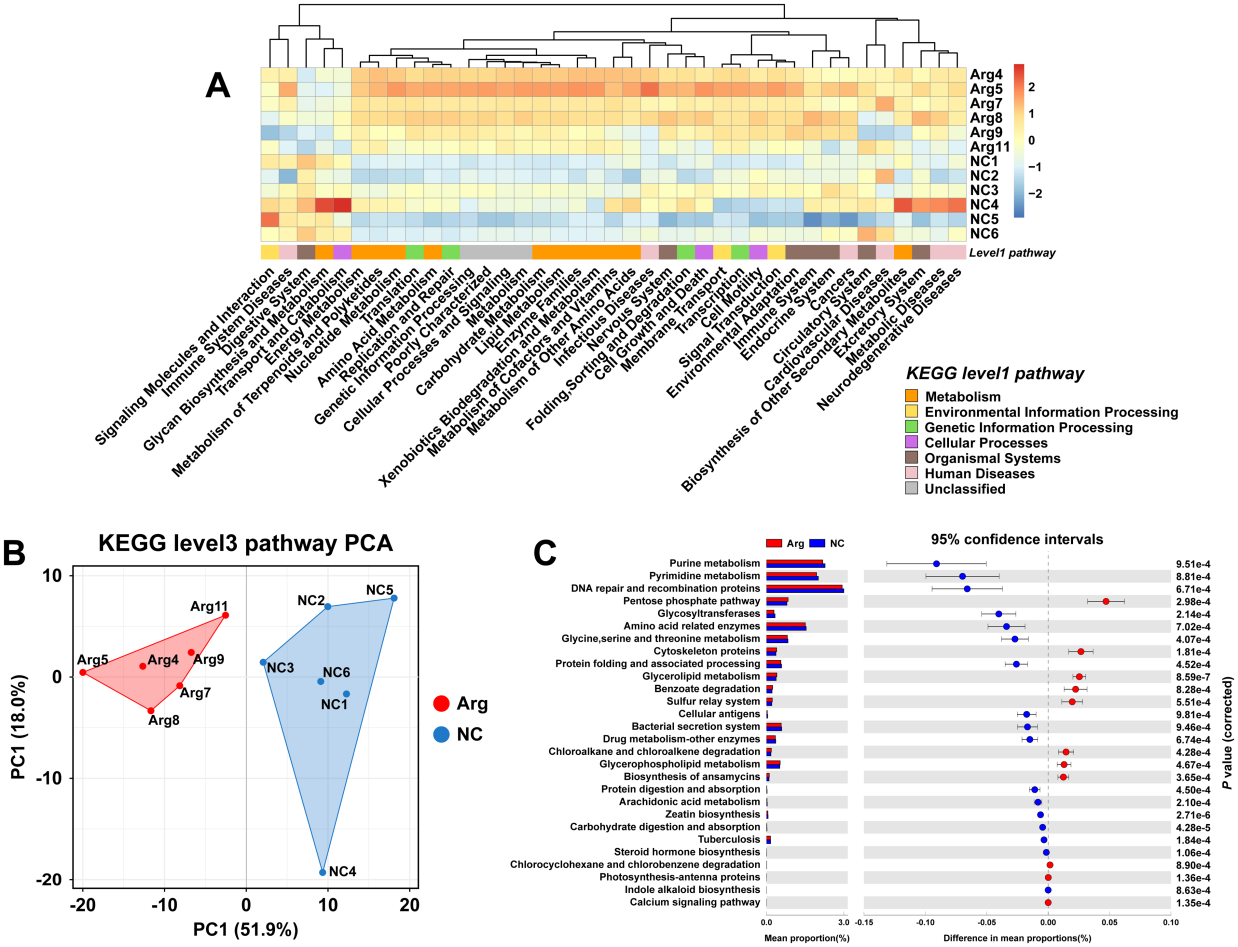
Functional prediction results of gut microbiota based on KEGG. **(A)** KEGG database level 1 and level 2 functional annotation heatmap. **(B,C)** PCA results and functional prediction differences for arginine group vs. control group in KEGG database level 3 pathways.

Using the KEGG database for functional prediction of 16S rRNA data, the results at the level 2 pathway reveal that most of the predicted pathways relate to metabolic functions (Figure 3A). PCA analysis also shows significant functional differences between the arginine-supplemented group and the control group (Figure 3B). Further investigation at the level 3 pathway prediction identifies 28 pathways with differences when *p* < 0.001, of which 18 pathways (64.29%) relate to metabolism (Figure 3C). These include pathways such as Nucleotide Metabolism, Amino Acid Metabolism, Carbohydrate Metabolism, Lipid Metabolism, Xenobiotics Biodegradation and Metabolism, Glycan Biosynthesis and Metabolism, Metabolism of Terpenoids and Polyketides, Energy Metabolism, and Biosynthesis of Other Secondary Metabolites. Notably, pathways related to Nucleotide Metabolism and Amino Acid Metabolism are primarily enriched in the control group, while pathways such as Energy Metabolism, Pentose Phosphate Pathway, and Glycan Biosynthesis and Metabolism are more prominent in the arginine-supplemented group. Additionally, within Lipid Metabolism and Xenobiotics Biodegradation and Metabolism, the arginine-supplemented group shows functional enrichment in Glycerophospholipid Metabolism, Glycerolipid Metabolism, Benzoate Degradation, Chloroalkane and Chloroalkene Degradation, and Chlorocyclohexane and Chlorobenzene Degradation.

### 3.3 Effect of feeding *L*-arginine on metabolites in the longest dorsal muscle of fattening pigs

**Figure 4:**
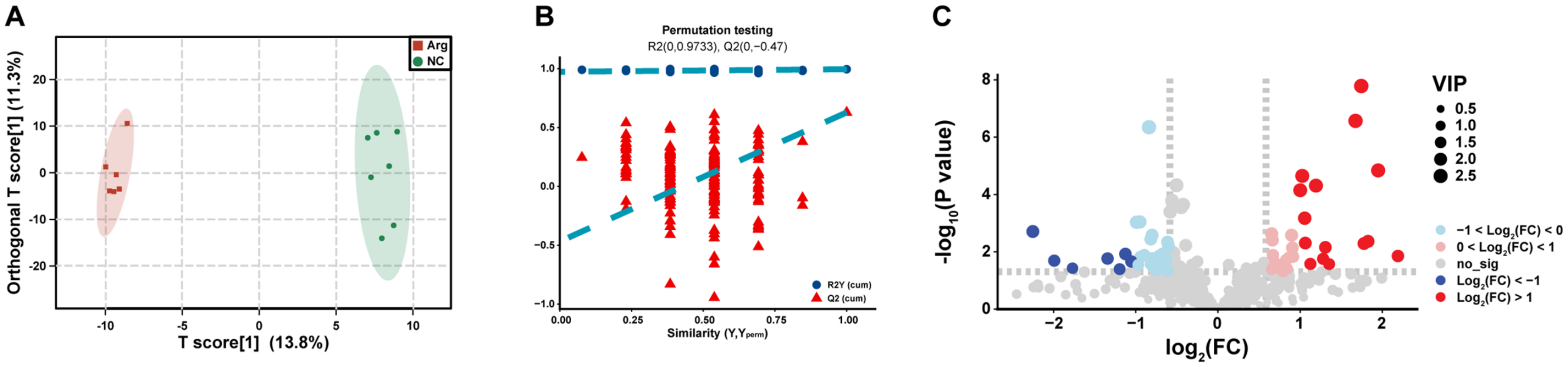
Differential metabolomics analysis between arginine group and control group. Panels **(A–C)** represent the OPLS-DA analysis results, permutation test results, and VIP-based differential volcano plot for the Arginine Group vs. Control Group, respectively.

Through OPLS-DA analysis, we observe complete separation in the T scores between the arginine-supplemented fattening pigs and the control group, indicating metabolic changes in the muscle of fattening pigs following arginine supplementation (Figure 4A). To mitigate false positive results, a permutation test is further conducted. The permutation test yields an *R*^2^*Y*(*cum*) of 0.995 and *Q*^2^(*cum*) of 0.628. Moreover, the intercepts of *R*^2^ and *Q*^2^ are 0.9733 and −0.047, respectively, demonstrating high model accuracy (Figure 4B). In summary, significant metabolic differences are found in the longest dorsal muscle of fattening pigs after arginine supplementation compared to the control group.

**Figure 5:**
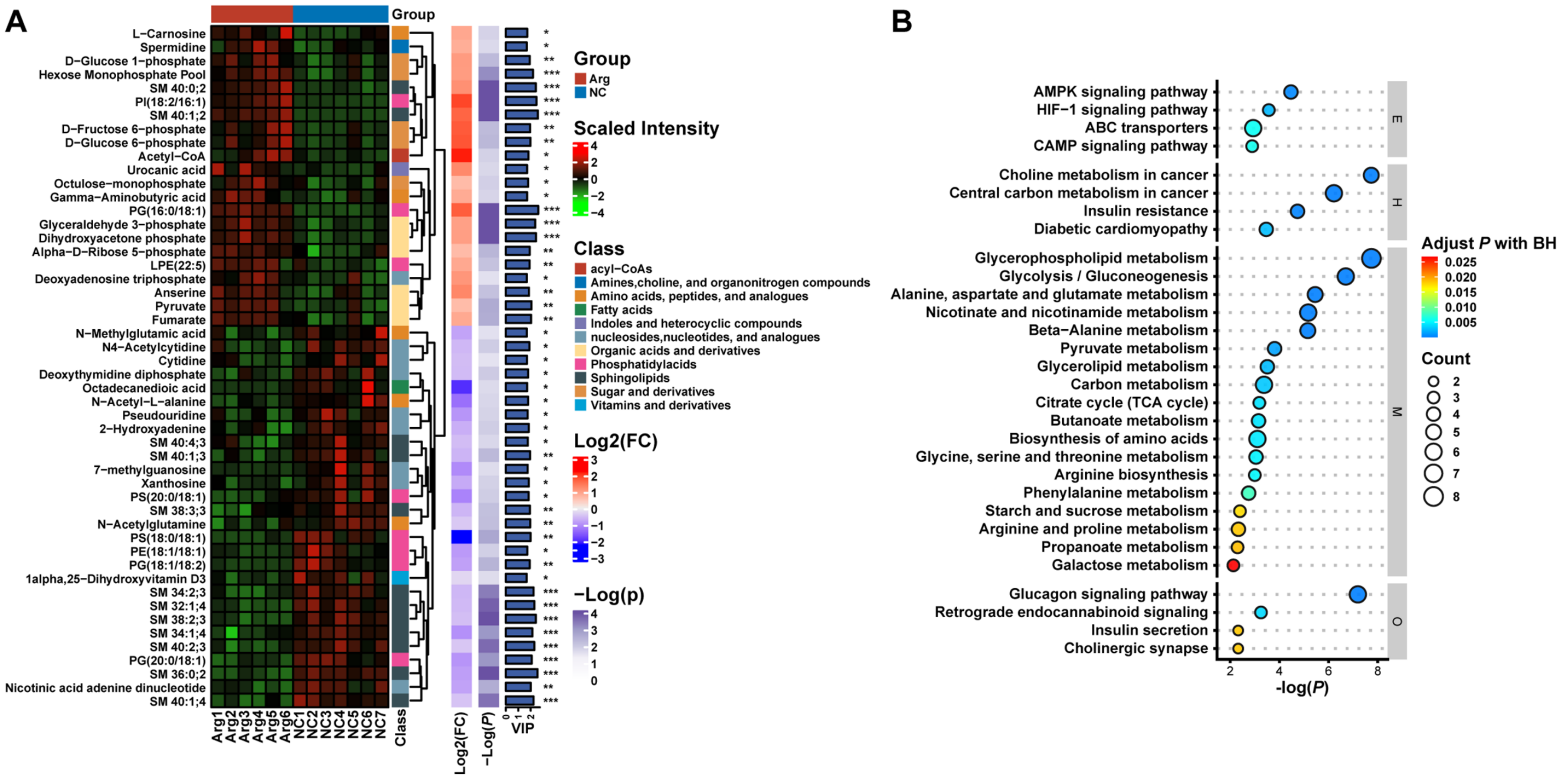
Multilayer clustering heatmap of differential metabolites **(A)** and functional annotation bubble plot **(B)**.

To delve deeper into the composition and impact of differential metabolites, we initially conduct a differential analysis based on the VIP values (volcano plot) (Figure 4C). With VIP > 1 and *p* < 0.05, a total of 85 differential metabolites are identified (Supplementary Table S1), comprising 38 upregulated and 47 downregulated metabolites (Supplementary Table S1). Subsequently, a cluster analysis (heatmap) is performed on the differential metabolites, categorizing them into approximately 11 classes, including Acyl-CoA, Amines, Choline and organic nitrogen compounds, Amino acids, Fatty acids, Sugars, and their derivatives. Post-arginine supplementation, an increase in abundance is noted in metabolites related to Organic acids and derivatives, as well as Indoles and heterocyclic compounds, while a decrease is observed in metabolites related to Sphingolipids and Fatty acids (Figure 5A). Finally, metabolites annotated with KEGG pathways undergo pathway enrichment analysis. The top 10 significantly enriched pathways include Glycerophospholipid metabolism, Choline metabolism in cancer, Insulin signaling pathway in cancer, Glycolysis/Gluconeogenesis, Central carbon metabolism in cancer, Alanine, aspartate and glutamate metabolism, Niacin and Niacinamide metabolism, *β*-Alanine metabolism, Insulin resistance, and the AMPK (AMP-activated protein kinase) signaling pathway (Figure 5B).

### 3.4 Correlation analysis between gut microbiota, metabolomics, and phenotypes

**Figure 6:**
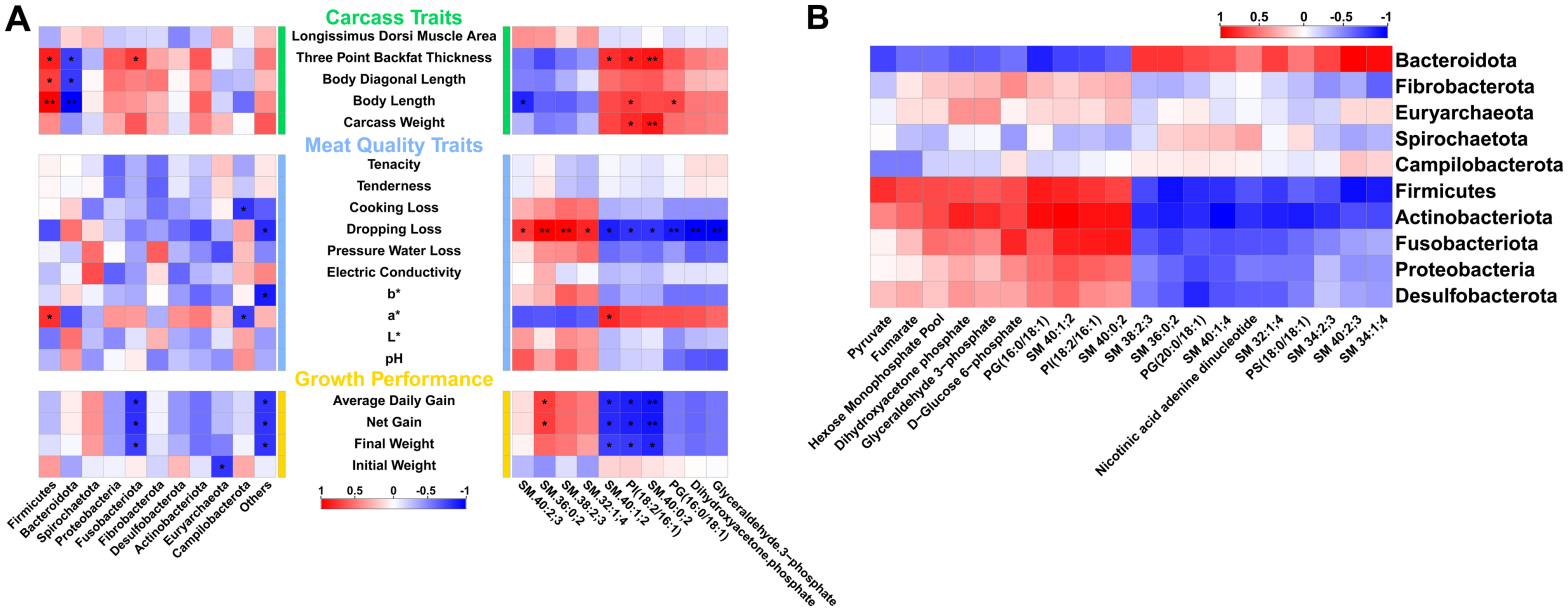
Correlation analysis between gut microbiota, metabolism, and phenotypes. **(A)** Correlation between gut microbiota, metabolomics, and different phenotypic traits. **(B)** Correlation between gut microbiota and differential metabolites.

Previous results indicate that arginine supplementation leads to varying degrees of changes in the phenotypes, gut microbiota composition, and metabolomics of fattening pigs. To explore the relationships among these three aspects, Pearson correlation analysis is conducted. The results show that Firmicutes and Bacteroidota, the two most significantly different phyla, are primarily associated with backfat thickness and body length. Firmicutes are positively correlated, while Bacteroidota are negatively correlated. Additionally, Fusobacteriota are significantly negatively correlated with growth performance traits such as Average Daily Gain, Net Gain, and Final Weight. Among the differential metabolites, the top 10 VIP metabolites are selected for correlation analysis. The results reveal that four sphingolipids—SM 40:2;3, SM 36:0;2, SM 38:2;3, and SM 32:1;4—demonstrate a positive correlation trend with most meat quality and growth performance traits, with drip loss being the most pronounced. All four sphingolipids show a significant positive correlation with drip loss, although they exhibit a negative correlation trend with some carcass traits. Conversely, the remaining six metabolites exhibit the opposite trend compared to the sphingolipids mentioned. Notably, two sphingolipids (SM 40:1;2 and SM 40:0;2) and one phosphatidic acid [PI(18:2/16:1)] are significantly negatively correlated with growth performance traits such as Average Daily Gain, Net Gain, and Final Weight (Figure 6A). Further correlation analysis between gut microbiota and the top 20 VIP metabolites from metabolomics reveals that five phyla, including Firmicutes and Actinobacteriota, are negatively correlated with the levels of most sphingolipids and phosphatidic acids, while showing a positive correlation with metabolites related to organic acids, sugars, and nucleotides. In contrast, Bacteroidota exhibit a completely opposite trend (Figure 6B).

## 4 Discussion

In livestock production, growth performance is a key indicator for evaluating animal productivity and economic efficiency (Tang et al., 2022; Goh et al., 2023). Arginine supplementation does not significantly impact growth performance metrics in finishing pigs, including final weight, net weight gain, and average daily gain. These results are consistent with those reported by Hu et al. (2017). Similarly, while carcass traits are crucial for assessing the meat value of livestock and hold significant economic importance, arginine supplementation does not enhance carcass quality in finishing pigs, which aligns with previous research on this subject (Ma et al., 2010). Meat quality traits serve as essential indicators for evaluating the quality of meat products, as they significantly influence consumers’ sensory experience and appetite (Aluwé et al., 2022). Experimental results show that arginine supplementation significantly increases the redness value (a*) of the longissimus dorsi muscle in finishing pigs 24 h post-slaughter. Previous studies demonstrate that adding arginine to daily feed markedly improves both the quantity and redness of oxidative muscle fibers in the biceps femoris of lambs (Dou et al., 2024). Similarly, Bao et al. (2021) observe that arginine treatment significantly enhances the redness of the longissimus dorsi muscle in both pork loin and pork belly. This suggests that arginine supplementation may increase the number of oxidative muscle fibers, which have higher myoglobin content, leading to a more vibrant color post-slaughter (Hnia et al., 2008; Jobgen and Wu, 2022). Additionally, this study finds that drip loss in the longissimus dorsi muscle is significantly lower in the arginine-supplemented group compared to the control group. Although not statistically significant, there is a noticeable trend toward reduced cooking and pressing losses in the longissimus dorsi muscle of finishing pigs following arginine supplementation. Previous research similarly reports that arginine supplementation can decrease cooking losses and enhance meat tenderness (Zhang Y. et al., 2020; Fan et al., 2024). After arginine supplementation, there is a significant increase in the abundance of Firmicutes in the gut microbiota of finishing pigs. Pearson correlation analysis further reveals a significant positive correlation between Firmicutes and the redness value (a*) of the longissimus dorsi muscle. This effect may be linked to lactobacilli, a component of Firmicutes, whose metabolic activity could reduce muscle pH and thereby affect the chemical state and color of myoglobin in the longissimus dorsi (O’Callaghan and O’Toole, 2013; Wang et al., 2021).

Next, we employ 16S rRNA sequencing to examine the impact of arginine supplementation on the gut microbiota of finishing pigs. Species diversity analysis reveals that arginine supplementation increases the abundance of gut microbiota and alters its structural composition, while not affecting the evenness of distribution. Consistent with previous studies, both the arginine and control groups show Firmicutes and Bacteroidetes as the predominant phyla. However, in the arginine group, there is a notable increase in Firmicutes abundance and a decrease in Bacteroidetes abundance (Sánchez et al., 2017; Zhang X.-Y. et al., 2023). Firmicutes, predominantly Gram-positive bacteria, play a crucial role in host nutrition and metabolism by producing short-chain fatty acids (Flint et al., 2008; Jordan et al., 2023; Kapoor et al., 2023; Sun et al., 2023). Bacteroidetes, often termed the “jack-of-all-trades” in the gut microbiota (Qu and Yuan, 2008; Hoyles and McCartney, 2009; Bolam and Sonnenburg, 2011), comprise various Gram-negative bacteria and are primarily involved in the digestion of dietary fiber polysaccharides and host glycans. In the functional predictions from PICRUSt2, the arginine group exhibits significant differences compared to the control group. At KEGG database levels 1 and 2, most of the divergent pathways relate to metabolism. Level 3 predictions further detail these metabolic differences, showing that arginine supplementation notably enhances pathways associated with gut microbiota energy metabolism, the pentose phosphate pathway, and glycerophospholipid metabolism. These results suggest that arginine supplementation impacts various functions and metabolic pathways within the gut microbiota of finishing pigs (Marini et al., 2017; Li et al., 2024).

The OPLS-DA analysis of the non-targeted metabolomics data from the longissimus dorsi muscle reveals a distinct separation between the arginine and control groups, indicating significant differences in endogenous metabolites. A total of 85 differentially expressed metabolites are identified, including amino acids, organic acids, and their derivatives, many of which act as substrates or intermediates in various metabolic processes. KEGG enrichment analysis highlights the top five most significantly enriched metabolic pathways: glycerophospholipid metabolism, choline metabolism in cancer, glucagon signaling pathway, glycolysis/gluconeogenesis, and central carbon metabolism in cancer. Glycerophospholipid metabolism, the most significantly enriched pathway, includes eight differentially expressed metabolites. Notably, 3-phosphoglycerate, an upregulated metabolite, serves as a crucial intermediate in fatty acid synthesis and as a precursor for cell membrane phospholipid synthesis (Saggerson and Greenbaum, 1970; Hamza et al., 2021). Glycerophosphocholine functions as both a storage form and a source of the neurotransmitter acetylcholine in the nervous system (Liu et al., 2022). Dihydroxyacetone phosphate plays a vital role in cellular metabolism, especially within the glycolysis and gluconeogenesis pathways (Underwood and Newsholme, 1967). In contrast, the downregulated metabolites—choline, phosphatidylcholine, phosphatidylethanolamine, and phosphatidylserine—primarily contribute to cell membrane composition and cell signaling processes (Volinsky and Kinnunen, 2013; Tayebati et al., 2017; Zhang L. et al., 2020; Whitlock and Chernomordik, 2021). Lysophosphatidylglycerol (18:2) primarily activates cell surface receptors, mediating various cellular responses such as migration, proliferation, and survival. It also participates in wound healing, angiogenesis, and immune responses (Jo et al., 2008; Subramanian et al., 2023). Therefore, we hypothesize that the effects of arginine supplementation on finishing pigs are primarily mediated through alterations in phospholipid metabolism, which subsequently influence the metabolism of various nutrients.

Finally, the correlation analysis between the gut microbiota and differential metabolites reveals that Firmicutes and Bacteroidetes, the primary phyla differing between the arginine and control groups, exhibit both positive and negative correlations with various phospholipid substances. This finding is further supported by Supplementary Table S2. A reduced abundance of Firmicutes and an increased abundance of Bacteroidetes in pigs serve as indicators of gut inflammation or cancer (Zhao et al., 2022). However, in the arginine group, these phyla show the opposite trend, suggesting that arginine supplementation alters the abundance of Bacteroidetes and Firmicutes in the gut of finishing pigs, thus helping to maintain intestinal homeostasis. Concurrently, these changes in gut microbiota structure also impact phospholipid metabolism, leading to altered phospholipid levels and, consequently, influencing certain phenotypes of the fattening pigs.

## 5 Conclusion

Supplementing the diet of finishing pigs with arginine does not significantly impact growth performance or carcass traits but improves meat quality. Arginine supplementation notably increases the redness value (a) of the longissimus dorsi muscle and significantly reduces drip loss, indicating improved appearance and tenderness of the pork. However, due to the short feeding period, the effects on other phenotypic traits remain limited. Additionally, arginine supplementation alters the gut microbiota and metabolome, characterized by an increased abundance of Firmicutes, a decreased abundance of Bacteroidetes, and reduced levels of most phospholipids. Correlation analysis reveals that the abundance of Firmicutes and Bacteroidetes closely links to phospholipid levels, with sphingolipids and phosphatidic acids strongly associated with changes in meat quality. This suggests that arginine supplementation affects the abundance of Firmicutes and Bacteroidetes in the gut, which in turn modifies phospholipid metabolism and ultimately influences meat quality. However, the specific molecular regulatory mechanisms require further investigation.

## Data Availability

The data presented in the study are deposited in the Genome Sequence Archive (GSA), accession number CRA019746.

## Footnotes

^1^http://www.lipidmaps.org/, accessed on 25 August 2022

^2^https://hmdb.ca/metabolites, accessed on 25 August 2022

^3^https://www.genome.jp/kegg/pathway.html, accessed on 25 August 2022
