# Supplementary material for: Effects of L-arginine on gut microbiota and muscle metabolism in fattening pigs based on omics analysis
Source: Front Microbiol. 2024 Nov 11;15:1490064. doi: 10.3389/fmicb.2024.1490064 (PMC11586382; doi:10.3389/fmicb.2024.1490064)
Supplement: Supplementary file 1 [file Table_1.DOCX]

| **序号 No.** | **代谢物名称**  **Metabolite** | **KEGG登录号**  **KEGG ID** | **VIP** | ***P* value** | **FC** |
| --- | --- | --- | --- | --- | --- |
| 1 | PG(16:0/18:1) |  | 2.610 | 1.64E-08 | 3.351 |
| 2 | SM 40:1;2 |  | 2.580 | 2.70E-07 | 3.192 |
| 3 | SM 36:0;2 |  | 2.553 | 4.50E-07 | 0.559 |
| 4 | PI(18:2/16:1) |  | 2.468 | 1.45E-05 | 3.865 |
| 5 | Dihydroxyacetone phosphate | C00111 | 2.431 | 2.25E-05 | 2.037 |
| 6 | SM 38:2;3 |  | 2.419 | 4.76E-05 | 0.705 |
| 7 | SM 40:0;2 |  | 2.415 | 4.91E-05 | 2.286 |
| 8 | Glyceraldehyde 3-phosphate | C00661 | 2.368 | 7.10E-05 | 2.002 |
| 9 | SM 32:1;4 |  | 2.299 | 1.634E-04 | 0.679 |
| 10 | SM 40:2;3 |  | 2.294 | 2.264E-04 | 0.746 |
| 11 | SM 34:2;3 |  | 2.233 | 4.015E-04 | 0.668 |
| 12 | SM 40:1;4 |  | 2.233 | 2.812E-04 | 0.733 |
| 13 | Hexose Monophosphate Pool |  | 2.186 | 0.001 | 2.079 |
| 14 | SM 34:1;4 |  | 2.141 | 0.001 | 0.503 |
| 15 | PG(20:0/18:1) |  | 2.082 | 0.001 | 0.518 |
| 16 | Pyruvate | C00022 | 2.065 | 0.002 | 1.573 |
| 17 | Fumarate | C00122 | 2.039 | 0.003 | 1.862 |
| 18 | Nicotinic acid adenine dinucleotide | C00857 | 2.006 | 0.003 | 0.574 |
| 19 | PS(18:0/18:1) | C02737 | 1.994 | 0.002 | 0.210 |
| 20 | D-Glucose 6-phosphate | C00092 | 1.985 | 0.004 | 3.548 |
| 21 | N-Acetylglutamine |  | 1.967 | 0.006 | 0.762 |
| 22 | D-Fructose 6-phosphate | C00085 | 1.955 | 0.005 | 3.429 |
| 23 | SM 40:1;3 |  | 1.949 | 0.005 | 0.653 |
| 24 | SM 38:3;3 |  | 1.942 | 0.006 | 0.659 |
| 25 | D-Glucose 1-phosphate | C00103 | 1.933 | 0.005 | 2.090 |
| 26 | Alpha-D-Ribose 5-phosphate | C03736 | 1.926 | 0.004 | 1.583 |
| 27 | PG(18:1/18:2) |  | 1.923 | 0.004 | 0.567 |
| 28 | LPE(22:5) |  | 1.913 | 0.007 | 1.874 |
| 29 | N4-Acetylcytidine | C22293 | 1.896 | 0.011 | 0.671 |
| 30 | Anserine | C01262 | 1.866 | 0.007 | 2.476 |
| 31 | 2-Hydroxyadenine |  | 1.849 | 0.012 | 0.626 |
| 32 | Xanthosine | C01762 | 1.849 | 0.014 | 0.609 |
| 33 | PS(20:0/18:1) |  | 1.838 | 0.012 | 0.458 |
| 34 | Acetyl-CoA | C00024 | 1.821 | 0.014 | 4.567 |
| 35 | Deoxythymidine diphosphate |  | 1.800 | 0.011 | 0.691 |
| 36 | N-Acetyl-L-alanine |  | 1.799 | 0.017 | 0.394 |
| 37 | Octulose-monophosphate |  | 1.796 | 0.013 | 1.590 |
| 38 | SM 40:4;3 |  | 1.773 | 0.018 | 0.693 |
| 39 | 7-methylguanosine |  | 1.761 | 0.022 | 0.481 |
| 40 | Pseudouridine | C02067 | 1.761 | 0.015 | 0.520 |
| 41 | PE(18:1/18:1) | C00350 | 1.729 | 0.013 | 0.537 |
| 42 | L-Carnosine | C00386 | 1.725 | 0.015 | 1.886 |
| 43 | Octadecanedioic acid |  | 1.722 | 0.020 | 0.251 |
| 44 | Gamma-Aminobutyric acid | C00334 | 1.720 | 0.016 | 1.819 |
| 45 | Urocanic acid | C00785 | 1.711 | 0.018 | 2.429 |
| 46 | Cytidine | C00475 | 1.705 | 0.029 | 0.697 |
| 47 | Deoxyadenosine triphosphate | C00131 | 1.686 | 0.026 | 2.182 |
| 48 | N-Methylglutamic acid |  | 1.685 | 0.026 | 0.578 |
| 49 | Spermidine | C00315 | 1.685 | 0.019 | 1.777 |
| 50 | 1alpha,25-Dihydroxyvitamin D3 | C01673 | 1.677 | 0.022 | 0.841 |
| 51 | Isovalerylglycine |  | 1.666 | 0.020 | 0.618 |
| 52 | Isovalerylcarnitine | C20826 | 1.662 | 0.034 | 1.707 |
| 53 | PG(16:0/18:2) |  | 1.661 | 0.019 | 0.646 |
| 54 | Docosatetraenoic acid |  | 1.651 | 0.017 | 0.574 |
| 55 | Glycerophosphocholine | C00670 | 1.633 | 0.028 | 1.095 |
| 56 | 4-Hydroxyphenylacetic acid | C00642 | 1.631 | 0.024 | 1.474 |
| 57 | L-Lactate | C00186 | 1.611 | 0.026 | 1.471 |
| 58 | CEA |  | 1.610 | 0.033 | 0.603 |
| 59 | 13S-hydroxyoctadecadienoic acid | C14762 | 1.603 | 0.027 | 2.556 |
| 60 | Citrulline | C00327 | 1.600 | 0.035 | 0.578 |
| 61 | L-2-Aminoadipic acid | C00956 | 1.600 | 0.040 | 0.436 |
| 62 | 1,5-anhydroglucitol | C07326 | 1.594 | 0.024 | 1.619 |
| 63 | Dimethylglycine | C01026 | 1.590 | 0.040 | 0.672 |
| 64 | FA(20:4) |  | 1.584 | 0.028 | 0.803 |
| 65 | Glucose | C00221 | 1.582 | 0.039 | 1.455 |
| 66 | LPA(18:2) | C00416 | 1.579 | 0.028 | 0.511 |
| 67 | Suberic Acid | C08278 | 1.575 | 0.026 | 0.580 |
| 68 | 1-Pyrroline-5-carboxylate | C03912 | 1.573 | 0.038 | 1.792 |
| 69 | n-Capric acid | C01571 | 1.567 | 0.030 | 0.673 |
| 70 | Hexose Pool |  | 1.564 | 0.041 | 1.457 |
| 71 | SM 36:1;2 |  | 1.563 | 0.044 | 1.712 |
| 72 | FA(20:3) |  | 1.561 | 0.032 | 0.727 |
| 73 | Guanosine | C00387 | 1.561 | 0.041 | 0.742 |
| 74 | 2-Naphthalenesulfonic acid | C16202 | 1.551 | 0.037 | 1.273 |
| 75 | PG(18:2/18:2) |  | 1.551 | 0.026 | 0.566 |
| 76 | Chenodeoxyglycocholic acid | C05462 | 1.550 | 0.041 | 0.802 |
| 77 | L-Argininosuccinic acid | C03406 | 1.533 | 0.040 | 1.577 |
| 78 | Choline | C00114 | 1.533 | 0.044 | 0.655 |
| 79 | GOLA |  | 1.523 | 0.045 | 0.595 |
| 80 | NAD | C00003 | 1.522 | 0.041 | 0.647 |
| 81 | 14,15-Epoxy-5,8,11-eicosatrienoic acid | C14771 | 1.505 | 0.048 | 1.730 |
| 82 | Glycerol 3-phosphate | C00093 | 1.501 | 0.035 | 1.375 |
| 83 | Tiglylglycine |  | 1.498 | 0.041 | 1.407 |
| 84 | PC(20:0/18:1) | C00157 | 1.465 | 0.037 | 0.293 |
| 85 | SM 38:0;2 |  | 1.398 | 0.044 | 1.340 |
