# Supplementary figures and images for: Effects of L-arginine on gut microbiota and muscle metabolism in fattening pigs based on omics analysis
Source: Front Microbiol. 2024 Nov 11;15:1490064. doi: 10.3389/fmicb.2024.1490064 (PMC11586382; doi:10.3389/fmicb.2024.1490064)

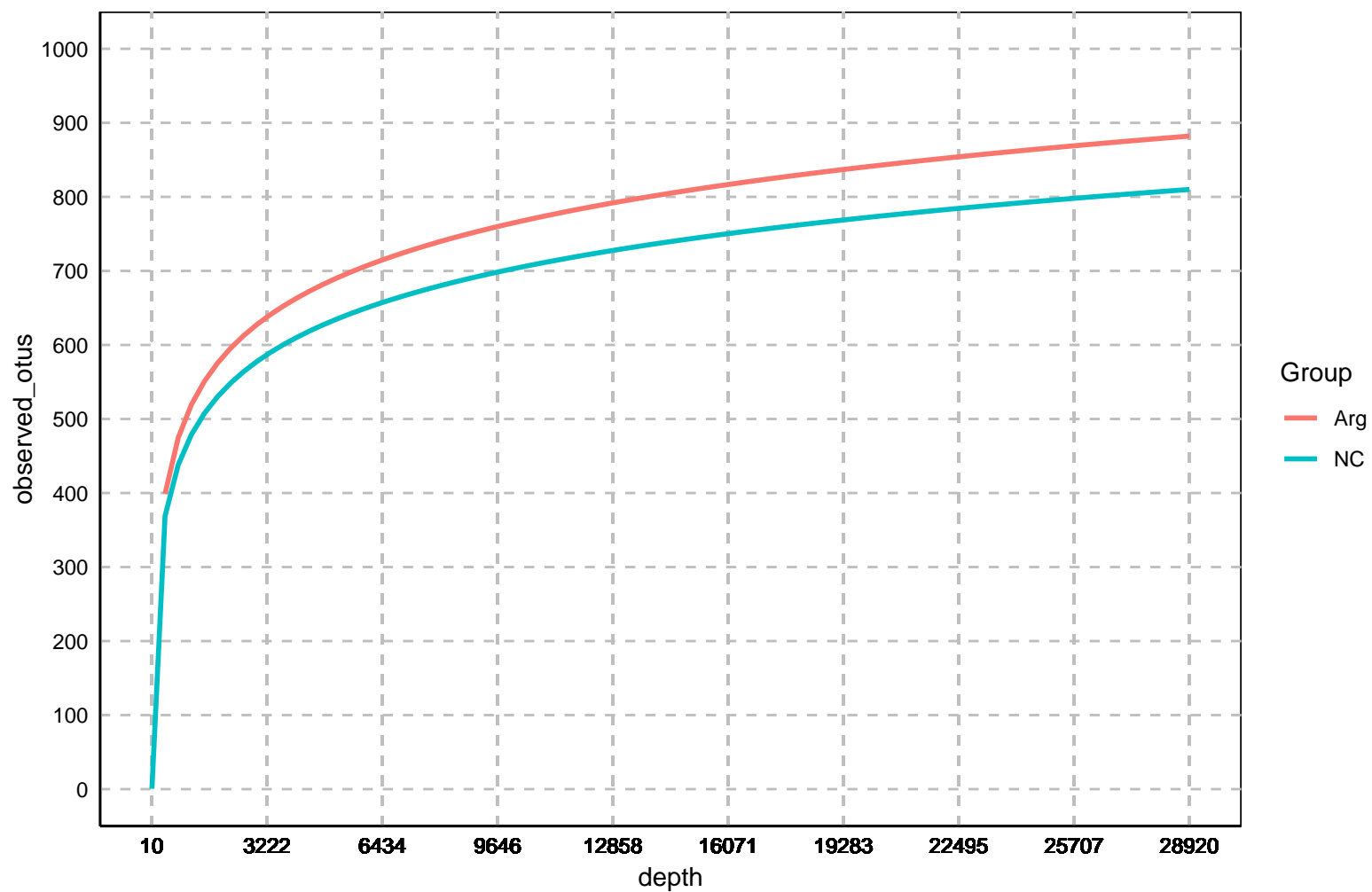

Supplement: Supplementary file 2 [file Data_Sheet_1.PDF]
